# *LRRK2* G2019S Mutation: Prevalence and Clinical Features in Moroccans with Parkinson's Disease

**DOI:** 10.1155/2017/2412486

**Published:** 2017-03-30

**Authors:** Ahmed Bouhouche, Houyam Tibar, Rafiqua Ben El Haj, Khalil El Bayad, Rachid Razine, Sanaa Tazrout, Asmae Skalli, Naima Bouslam, Loubna Elouardi, Ali Benomar, Mohammed Yahyaoui, Wafa Regragui

**Affiliations:** ^1^Research Team in Neurology and Neurogenetics, Medical School and Pharmacy, Mohammed V University, Rabat, Morocco; ^2^Department of Neurology and Neurogenetics, Specialties Hospital, Rabat, Morocco; ^3^Laboratory of Public Health, Medical School and Pharmacy, Mohammed V University, Rabat, Morocco

## Abstract

*Background*. The* LRRK2* G2019S mutation is the most common genetic determinant of Parkinson's disease (PD) identified to date. This mutation, reported in both familial and sporadic PD, occurs at elevated frequencies in Maghreb population. In the present study, we examined the prevalence of the G2019S mutation in the Moroccan population and we compared the motor and nonmotor phenotype of G2019S carriers to patients with idiopathic Parkinson's disease.* Methods*. 100 PD patients were assessed for motor and nonmotor symptoms, current medication, and motor complication including motor fluctuations and dyskinesia. The* LRRK2* G2019S mutation was investigated by direct sequencing in patients and ethnically matched controls, all of Moroccan origin.* Results.* Among the 100 PD Moroccan patients, 41 (41%) were carriers of the G2019S mutation. The mutation frequency was higher among probands with autosomal dominant inheritance (76%) than among sporadic ones (28%). Interestingly, G2019S mutation was also found in 5% of control individuals. Clinically, patients carrying the G2019S mutation have more dystonia (OR = 4.6,* p* = 0.042) and more sleep disorders (OR = 2.4,* p* = 0.045) than noncarriers.* Conclusions.* The* LRRK2* G2019S prevalence in Morocco is the highest in the world reported to date. Some clinical features in G2019S carriers such as dystonia and sleep disturbances are worth noting.

## 1. Introduction

Parkinson's disease (PD) is the second most common neurodegenerative disorder after Alzheimer's disease affecting approximately 1-2% of the population over 60 years and 4% above 85 years [1]. It is clinically characterized by rigidity, bradykinesia, tremor, and postural instability. Other clinical features as dementia and depression can be added to this clinical array [2, 3]. Pathologically it is identified by a selective degeneration of dopaminergic neurons in the substantia nigra in the midbrain and eventually the presence of Lewy bodies in the surviving neurons [4, 5]. The etiology of PD is likely to be multifactorial involving complex interactions between genetic and environmental factors, but the exact molecular mechanism underlying the pathogenesis of the disease remains obscure. In the past 17 years, genetic studies of PD families consolidate the hypothesis that PD has a significant genetic component. Indeed, 14 genes have been described for Mendelian PD so far [6, 7]. Among them, there are at least three confirmed genes responsible for the autosomal dominant form of PD:* SNCA* (PARK1/4),* LRRK2* (PARK8), and* VPS35* (PARK17).

Mutations in* LRRK2* gene are the most frequently reported monogenic cause of PD and are common in both early and late-onset PD, occurring in both familial and sporadic PD patients with a wide variety of clinical and pathological features and a variable frequency depending on ethnic origin [8]. Among these mutations, the glycine to serine substitution (G2019S), located within the protein kinase domain encoded by exon 41, is the most common and was estimated by the international* LRRK2* consortium to represent 1% of sporadic and 4% of familial PD patients worldwide [9]. Intriguingly, the frequency of this mutation varies greatly among ethnic groups and geographic origins. In fact, the highest frequencies were observed in North African countries with 30–40% and Ashkenazi Jews with 10–30% [10, 11]. In Europe, the frequency of G2019S mutation appears to be relatively higher in southern countries particularly in Portugal and Spain with 2–14% of PD cases, than in northern countries with 0–3% [9–13] suggesting a European north-south gradient. The presence of G2019S in PD patients is very rare in Asian populations with a frequency less than 0.1% in China, Japan, Korea, and India, whereas it can reach 1–3% in white North American population [8, 10, 11, 14]. However, none of black PD patients from Nigeria and South Africa seems to carry the G2019S mutation [15, 16]. Among apparently healthy controls, the highest frequency of the G2019S mutation has been reported in North Africa with 3.3% in Berbers of Morocco, 2.13% in Algerians, 1.57% in Tunisians, and 1.32% in Libyans [17]. This frequency is estimated at 2% in Ashkenazi Jews [18] and is reported as very rare or absent in other populations [9]. Occurrence of the G2019S mutation in patients with PD and healthy subjects suggests reduced penetrance, which has been shown to vary according to ethnic origin [12, 19–22]. This variability in penetrance suggests that other genetic or environmental factors are involved in the pathogenesis of the disease.

Clinically, G2019S mutation carriers develop a very similar PD disease to noncarriers, including the development of motor symptoms and cognitive impairment [9, 23], but some differences could be observed even within the same family [24]. Homozygous carriers of G2019S mutation are rare, mostly reported in North African populations where the rate of consanguineous marriages is high. These patients do not show differences in clinical features compared with heterozygous carriers [25–30].

The present study aims to estimate the prevalence of the G2019S mutation of the* LRRK2* gene in the Moroccan population and to assess the motor and nonmotor phenotype of G2019S mutation carriers and noncarriers.

## 2. Subjects and Methods

A total of 100 unrelated PD patients were recruited consecutively from the outpatient clinic of the Neurological Department at University Hospital Ibn Sina of Rabat from October 2013 to June 2015. One hundred healthy individuals were recruited from the National Blood Transfusion Institute of Rabat and were used as controls. Their mean age was 58.59 (±8.65) and 51 of them were males. Patients and controls provided a written informed consent and the study was approved by the ethic committee of the Medical School of Rabat (CERB).

### 2.1. Clinical Evaluation

Diagnosis of PD was made by the same neurologist using the United Kingdom Parkinson's Disease Society Brain Bank criteria [31]. Patients were submitted to a structured clinical interview including demographic data, date of onset, disease duration, motor phenotype subtype, the presence of dystonia in the early disease course, motor fluctuations and dyskinesia, nonmotor symptoms, and current medication. Motor symptoms were assessed using the Unified Parkinson's Disease Rating Scale part III and Hoehn and Yahr stage during ON condition. The LEDD (levodopa Equivalent Daily Dose) was calculated based on a previously published algorithm combining dopamine agonist daily dose with levodopa daily dose [32]. We classified the motor phenotype as tremor-dominant, akinetic-rigid, or mixed, and for the purposes of analysis owing to low figures, we included akinetic-rigid phenotype in mixed group. Nonmotor symptoms scales used are the Pittsburgh sleep QI for sleep disturbances, the Epworth Sleepiness Scale for excessive daytime sleepiness, the SCOPA autonomic questionnaire for dysautonomia, the DN4 questionnaire for neuropathic pain, the Hamilton and the Montgomery and Asberg Depression Rating Scale (MADRS) for depressive complaints, and the Arab version of the MMSE for cognitive impairment (patients with scores below 21 were excluded to avoid reliability concerns in their answers relative to the scales of the questionnaire). For simplification, we recorded nonmotor symptoms as absent or present for constipation, urinary urgency, orthostatic vertigo, pain, hallucinations, memory complaints, and sleep disturbances.

Otherwise, all control individuals have no family history of neurological disease but have not been clinically assessed for the presence of PD.

### 2.2. Genetic Analysis

A pedigree was established for all patients and the mode of inheritance was classified as “familial” if at least one relative was reported with a diagnosis of PD (FPD) and as autosomal recessive or dominant based on the presence or absence of consanguinity. The remaining patients were classified as “sporadic” (SPD).

Genomic DNA was extracted from peripheral blood leukocytes using Isolate II Genomic DNA kit from Bioline. The G2019S mutation of the* LRRK2* gene was performed by direct sequencing. Briefly, a 378 bp* LRRK2* exon 41 fragment was PCR amplified as described previously [33]. The PCR products were sequenced using Big Dye Terminator Cycle Ready Reaction 3.1 Kits and an ABI 3130xl automated sequencer, and sequence chromatograms were analyzed using SeqScape 2.1 software (Applied Biosystems, Foster City, CA).

### 2.3. Statistical Analysis

Demographic and clinical variables between G2019S-carriers and noncarriers were compared using parametric and nonparametric tests as appropriate using SPSS 13.0 software. Quantitative data were expressed in mean ± standard deviation (SD) or median and interquartile range and were compared using *t*-test or Mann–Whitney test. Categorical variables were expressed as numbers and percentages and were compared using Chi-square test. The relationship between G2109S mutation and the clinical symptoms was analyzed by means of logistic regression adjusting for age, sex, and disease duration on univariate and multivariate analysis. In the multivariate analysis model, we introduced variables that had a* p* ≤ 0.3 and we forced this analysis for Hoehn and Yahr score for its importance in the disease evolution.* p* value < 0.05 was considered as statistically significant. For multiple testing, we corrected the* p* value by the Bonferroni method.

## 3. Results

We examined 100 patients with PD, 56 of whom were males and 44 were females (Table 1). The mean age at exam was 60.93 (±11.07) and the mean age at onset was 53.9 years (±11.54). Comparison of demographic features between the G2019S carriers and noncarriers (Table 1) showed no significant difference except for the disease duration; G2019S carriers have the longest disease duration (*p* = 0.03).

### 3.1. Genetic Aspects

Sixty-seven out of 100 patients were sporadic cases (67%) and 33 had a positive family history of PD (33%). Ten of the 67 SPD patients were from consanguineous marriages (Table 2). Among the 33 FPD patients, 29 had dominant inheritance (DFPD), 2 had recessive inheritance (RFPD), and in 2 patients the mode of inheritance could not be specified (parents were consanguineous and one of them is with PD). The* LRRK2* G2019S substitution was found in 41 of 100 (41%, 95% CI 31.4–50.3) PD patients, 37 of whom were heterozygous and 4 were homozygous. The G2019S prevalence increased to 67% (95% CI 48.11–81.45) for FPD patients, with 76% of patients (95% CI 56.07–88.98) having a DFPD, 0% of patients (0 of 2) having RFPD, and 0% of patients (0 of 2) having an unspecified mode of inheritance. The prevalence reaches only 28% (95% CI 18.35–40.88) for SPD patients with 32% of patients (95% CI 20.27–45.38) without consanguinity and 10% of patients (95% CI 1.79–40.41) with consanguinity (Table 2). Interestingly, there were five control individuals heterozygous for the G2019S mutation among the 100 tested (5%, 95% CI 1–10).

### 3.2. Clinical Features

Motor and nonmotor symptoms of all patients are given in Table 3. The initial symptom of the disease and the clinical phenotype were significantly different between LRRK2 G2019S carriers and noncarriers (*p* = 0.019 and* p* = 0.012, resp.). LRRK2 G2019S carriers have less tremor than noncarriers do as first symptom (26.8% versus 52.5%, corrected-*p* = 0.03) and exhibited less of the tremor-dominant phenotype than noncarriers (corrected-*p* = 0.009). They also have more dystonia (*p* = 0.011) and more dyskinesia (*p* = 0.002) and take a higher dose (up to 200 mg) of dopaminergic drugs (*p* = 0.002). Gait disturbances, postural instability, motor fluctuations, UPDRS III score, and H&Y scale during ON state are similar between both groups. Concerning nonmotor symptoms,* LRRK2* G2019S carriers have more sleep complaints than noncarriers do (*p* = 0.046), while they show the same rates of psychiatric symptoms, constipation, urinary urgency, and orthostatic vertigo. There is a trend to more cognitive impairment in* LRRK2* G2019S carriers than noncarriers but the difference is not significant (*p* = 0.059).

The relationship between G2109S mutation and the clinical symptoms using logistic regression by adjusting for age, sex, and disease duration is shown in Table 4.* LRRK2* G2019S mutation is associated with more dystonia (OR = 4.655,* p* = 0.042) and sleep complaints (OR = 2.4,* p* = 0.045) but less tremor (OR = 0.3,* p* = 0.011). Nonetheless, while* LRRK2* G2019S carriers have more levodopa-induced dyskinesia (*p* = 0.002), the statistical significance lacked on multivariate analysis (OR = 1.965, *p* = 0.217).

## 4. Discussion

The international* LRRK2* consortium reported a worldwide frequency of* LRRK2* G2019S mutation of 1% in sporadic PD and 4% in familial PD [9]. Although the highest frequency of the G2019S mutation has been recorded in North Africa, no study on the prevalence of this mutation in the Moroccan population has been conducted until now. Previous studies have used small sample size of Moroccan patients, the majority of whom were living outside Morocco. In our cohort of 100 PD patients of Moroccan origin, the overall mutation frequency of G2019S is 41% (95% CI 31.4–50.3). Among probands with autosomal dominant mode of inheritance, this value rises to about 76% (95% CI 56.07–88.98) and 28% (95% CI 18.35–40.88) among sporadic cases. Unexpectedly, these frequencies were higher than observed in PD patients from neighboring Maghreb countries such as Algeria and Tunisia [10, 11], representing the highest prevalence in the world reported to date for the G2019S mutation.

Among the 41 patients with G2019S mutation, four were homozygous carriers with different mode of inheritance, including two with autosomal dominant inheritance and two sporadic cases of which one comes from a consanguineous marriage. This could be due to the high prevalence of the mutation in the general population. Indeed, there were five heterozygous G2019S carriers among the 100 control individuals (5%).

Clinically, most authors reported a similar phenotype between* LRRK2* G2019S carriers and noncarriers [34, 35]. In our series, the phenotypes are overlapping but the* LRRK2* subjects have less tremor. This finding is in line with some series [34] whereas others reported the tremor as a more common presenting feature in* LRRK2* carriers [30, 36, 37]. Contrariwise, we found a nonsignificant increase of dyskinesia frequency as reported earlier [38], which can be explained by the higher LEDD recorded in this group. Moreover,* LRRK2* G2019S carriers had a similar UPDRS III and H&Y scores for longest disease duration (7 [4–13] versus 5 [2–7],* p* = 0.035) compared to noncarriers. It can reflect in some extent a slower disease evolution in LRRK2 G2019S patients. In the same line, Healy et al. [9] reported longer latency between disease onset and first fall among carriers compared to noncarriers. However, longitudinal follow-up is needed to compare disease course between the two groups.

Furthermore, dystonia is the most specific feature that characterizes* LRRK2* G2019S carriers in our series with an ODDS ratio of 4.65. Dystonia is a well-defined symptom in early onset Parkinson's disease (EOPD) as reported by Kilarski et al. [39] in their systematic review and UK-based study of EOPD. The high frequency in our study can be explained somehow by the overall young age of onset of Parkinson's disease in Moroccan patients [40], but there was no statistically difference in age of onset between* LRRK2* G2019S carriers and noncarriers (*p* = 0.207).

Otherwise,* LRRK2* G2019S patients exhibited more sleep complaints in our series. This feature is in line with other series that reported more sleep onset insomnia, few or no rapid eye behavior disorders, and less nocturia in* LRRK2* G2019S carriers [41–43].

Other reports described more specific clinical features in* LRRK2* carriers such as a lower limb onset [34], more hallucinations, behavioral disorders, and dopaminergic dysregulation syndrome [36, 38, 44] but less cognitive impairment [34, 36, 45]. Kalia et al. [46] explained this phenomenon by the lack of Lewy bodies in some cases with* LRRK2* G2019S mutation; the presence of Lewy bodies is strongly correlated to some nonmotor symptoms especially cognitive impairment and dementia. In our study,* LRRK2* carriers have a trend to more cognitive impairment that was not confirmed in the logistic regression model. Future studies with wider cohorts are required to determine the cognitive profile of* LRRK2* carriers.

## 5. Conclusions

Our study showed that Morocco has the highest reported prevalence of the G2019S mutation in the world, with a mutation frequency of 41% overall and 76% for patients with autosomal dominant mode of inheritance. Furthermore, G2019S carrier patients exhibit clinical features quite similar to noncarriers with some mild differences in particular more dystonia and more sleep complaints.

## Figures and Tables

**Table 1:** Demographic features of LRRK2 G2019S carrier and noncarrier patients.

Variables	All patients	G2109S carriers	G2019S noncarriers	*p* value
	(*n* = 100)	(*n* = 41)	(*n* = 59)	
Age at exam (years)^a^	60.93 ± 11.07	60.32 ± 11.83	61.35 ± 10.59	0.649
Age at onset (years)^a^	53.9 ± 11.54	52.15 ± 11.28	55.12 ± 11.65	0.207
Disease duration (years)^b^	6 [2.25–10]	7 [4–13]	5 [2–7]	0.035
Gender (male)^c^	56	19 (46.3)	37 (62.7)	0.105

Data are ^a^mean  ± standard deviation, ^b^median [interquartile range], and ^c^numbers (percentage).

**Table 2:** Prevalence of G2019S mutations among 100 Moroccan PD patients.

Form	Familial				Sporadic cases		
Inheritance	DFPD	RFPD	ND	Total	Inbred	Not inbred	Total
Patients	29 (29)	2 (2)	2 (2)	33 (33)	10 (10)	57 (57)	67 (67)
G2019S carriers	22 (76)	0 (0)	0 (0)	22 (67)	1 (10)	18 (32)	19 (28)

Data are number (percentage). DFPD = dominant familial Parkinson's disease, RFPD = recessive familial Parkinson's disease, and ND = inheritance not determined.

**Table 3:** Clinical features of G2019S carrier and noncarrier patients.

	G2109S carriers	G2019S noncarriers	*p *value
	(*n* = 41)	(*n* = 59)	
Initial symptom			**0.019**
Akinesia^a^	14 (34.1)	9 (15.3)	
Tremor^a^	11 (26.8)	31 (52.5)	
Tremor and akinesia^a^	16 (39)	19 (32.2)	
Clinical phenotype			**0.012**
Akinetic-rigid^a^	10 (24.4)	10 (16.9)	
Tremor-dominant^a^	7 (17.1)	27 (45.8)	
Mixed^a^	24 (58.5)	22 (37.3)	
Dystonia^a^	9 (21.9)	3 (5.1)	**0.011**
Gait impairment^a^	21 (51.2)	23 (39)	0.225
Postural instability^a^	17 (41.5)	29 (49.2)	0.448
UPDRS III ON^b^	11 [6.5–19]	11 [6–21]	0.858
H-Y score^b^	2 [1–4]	3 [1–4]	0.875
LEDD^b^	727.9 [500–1100]	500 [300–800]	**0.002**
Fluctuations^a^	25 (61)	29 (49.2)	0.243
Dyskinesia^a^	23 (56.1)	15 (25.4)	**0.002**
Urinary dysfunction^a^	30 (73.2)	48 (82.8)	0.253
Constipation^a^	21 (51.2)	31 (52.5)	0.896
Orthostatic hypotension^a^	16 (39.0)	29 (49.2)	0.318
Pain^a^	22 (53.7)	31 (52.5)	0.912
Psychiatric disorders^a^	25 (61.0)	28 (47.5)	0.184
Cognitive disorders^a^	26 (63.4)	26 (44.1)	0.059
Sleep disorders^a^	22 (53.7)	19 (33.3)	**0.046**

Data are ^a^number (percentage) and ^b^median [interquartile range]. Significant *p *values are in bold.

**Table 4:** Logistic regression model of the association between G2019S mutation and PD clinical features adjusted for gender, age, and disease duration.

	OR/*β*	CI 95%	*p *value
Initial symptom	0.305	0.123–0.758	0.011
Tremor^a^			
Akinesia + mixed^a^			
Clinical phenotype	0.261	0.096–0.708	**0.008**
Tremor-dominant^a^			
Akinetic-rigid + mixed^a^			
Dystonia^a^	4.655	1.058–20.48	**0.042**
H-Y score^b^	−0.104	−0.666–458	0.717
LEDD^b^	52.57	−103.6–208.7	0.509
Fluctuations^a^	0.606	0.206–1.782	0.363
Dyskinesia^a^	1.965	0.673–5.739	0.217
Urinary dysfunction^a^	0.566	0.207–1.550	0.268
Psychiatric disorders^a^	2.023	0.856–4.782	0.109
Cognitive disorders^a^	1.892	0.812–4.409	0.140
Sleep disorders^a^	2.409	1.021–5.685	**0.045**

Data are ^a^numbers (percentages) and ^b^median [interquartile range]. OR = odds ratio and CI = confident interval. Significant *p *values are in bold.
